# Re-growth of an incomplete discoid lateral meniscus after arthroscopic partial resection in an 11 year-old boy: a case report

**DOI:** 10.1186/1471-2474-14-285

**Published:** 2013-10-07

**Authors:** Salvatore Bisicchia, Cosimo Tudisco

**Affiliations:** 1 Department of Orthopaedic Surgery, University of Rome “Tor Vergata”, Viale Oxford 81, Rome 00133, Italy

**Keywords:** Discoid meniscus, Arthroscopy, Re-growth, Morphogenesis

## Abstract

**Background:**

Discoid lateral meniscus is common in children. Arthroscopic partial resection is indicated in symptomatic cases generally achieving satisfactory results.

**Case presentation:**

We present a case of an incomplete discoid lateral meniscus of the right knee in an 11 year-old boy, treated with arthroscopic partial resection, which developed a re-growth of the remnant, restoring the pre-operative incomplete discoid shape. To the best of our knowledge this is the first report about re-growth of a discoid meniscus after surgery. Debate still exists regarding the etiology of a discoid meniscus. Some authors proposed it is the persistence of the normal stage during fetal development. However, most other authors believe it is anomalous and arises through variant morphogenesis. The re-growth of the discoid lateral meniscus following surgery in this patient seems to prove this latter theory. The residual growth of the knee involves also the lateral meniscus and that may have contributed to restoring the meniscus to the previous condition.

**Conclusion:**

This case report demonstrates discoid meniscal re-growth in a child. The growth spurt may have an impact on meniscal regeneration. Re-growth of the discoid lateral meniscus in our patient favors the hypothesis of variant morphogenesis.

## Background

The prevalence of discoid lateral meniscus ranges from 0.4 to 17% and it is bilateral in up to 20% of cases. Surgical treatment is indicated in symptomatic cases, with satisfactory results following arthroscopic partial resection [1]. Discoid meniscus can be classified as complete, incomplete and Wrisberg types [2].

We present a case of an 11 year-old boy, arthroscopically treated for an incomplete discoid lateral meniscus of the right knee that developed a re-growth of the remnant, restoring the pre-operative incomplete discoid shape. To the best of our knowledge this is the first report regarding re-growth of a discoid meniscus after surgery.

## Case presentation

**Figure 1:**
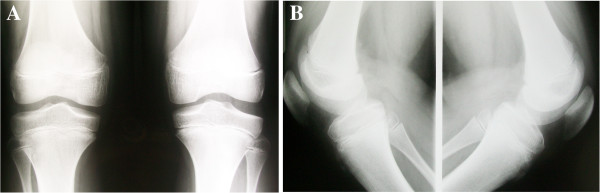
Antero-posterior (A) and lateral (B) standing X-rays showing bilateral squaring of the lateral femoral condyle and hypoplasia of the lateral tibial spines.

**Figure 2:**
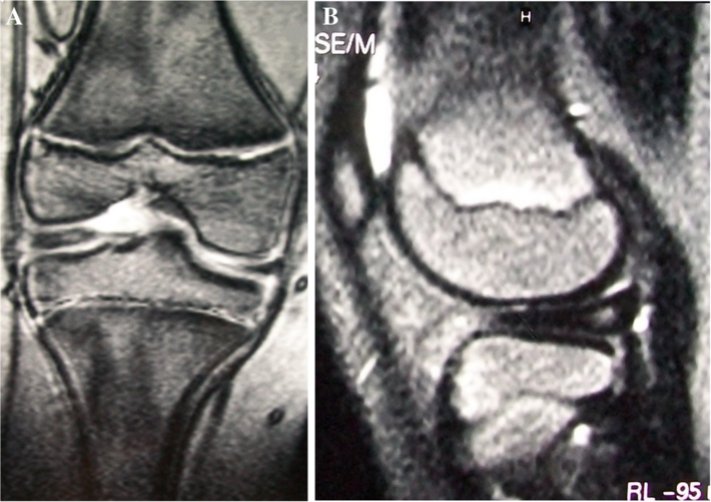
Coronal (A) and sagittal (B) MRI views of the right knee performed when the patient was 11 year-old showing an incomplete discoid lateral meniscus.

A 11-year-old boy presented with a three-month history of recurrent pain on the right knee with squatting, jumping and swimming breaststroke style. He did not report any trauma. In the last several years, he also complained about recurrent painless “clunks” on both knees especially during squatting. He used to practice martial arts, but he had to stop because of the pain. At physical examination both knees were not swollen and showed a complete and painless range of motion. A “clunk” was audible and it was visible and palpable during active and passive motion. McMurray test was negative. Left knee scored fair and right knee scored poor according to Ikeuchi scale [3]. X-rays showed squaring of the lateral femoral condyle and hypoplasia of the lateral tibial spine bilaterally (Figure 1). MRI revealed bilateral incomplete discoid lateral meniscus with normal medial menisci and open physes (Figure 2).

**Figure 3:**
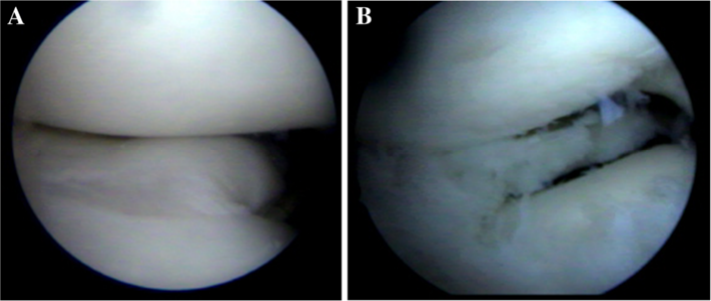
Arthroscopic photographs of the right knee taken during the first surgery showing incomplete discoid lateral meniscus (A) and a 8 mm functional rim after partial resection (B).

To relieve pain and restore function, arthroscopic partial resection of the lateral meniscus of the right knee was performed by the senior author, who is trained in pediatric knee arthroscopy, leaving a functional residual rim of 8 mm (Figure 3). Physical therapy was begun immediately with isometric exercises, partial to total weight bearing was allowed as tolerated. The postoperative course was uneventful, after 2 months the patient returned to sports activities without any restriction and was followed-up clinically at 1, 2, 3, 6 and 12 months after surgery and then yearly.

**Figure 4:**
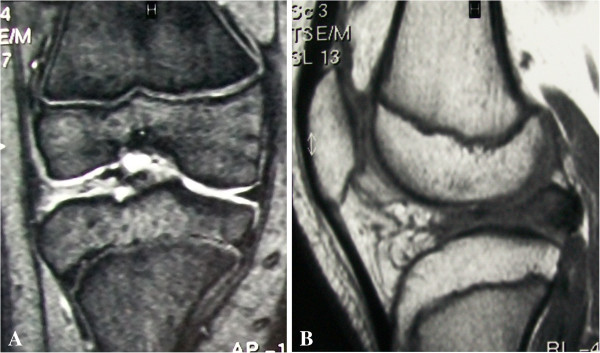
**Coronal (A) and sagittal (B) MRI views of the right knee at 29 months follow-up.** Note the discoid shape of the lateral meniscus in both coronal and sagittal views. The re-grown meniscus showed a different signal at MRI compared with the native one. Growth plates were still visible.

**Figure 5:**
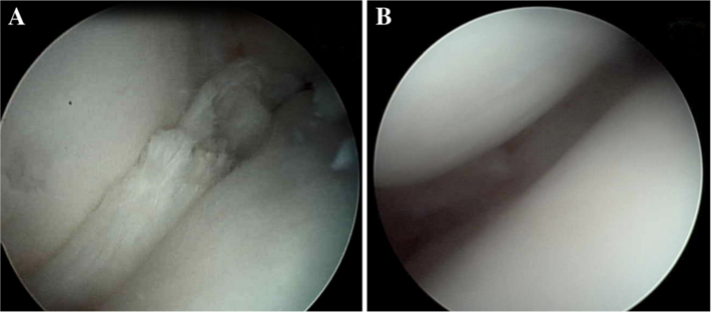
Arthroscopic photographs of the right knee taken during second surgery showing re-growth of an incomplete discoid lateral meniscus (A) and a 8 mm functional rim after partial resection (B).

Twenty-nine months after surgery the patient returned to our clinic for a non-scheduled follow-up visit complaining about the same symptoms as before surgery, again only on the right knee. The onset of the symptoms was subtle and the patient did not report any trauma. In the meantime, he was going through a growth spurt and he had grown about 15 cm since the first surgery. MRI of the right knee showed an incomplete discoid lateral meniscus, with a different signal intensity compared with the original one, and open physis (Figure 4). Arthroscopy of the right knee was done again by the same surgeon, confirming the discoid re-growth of the lateral meniscus, with a horizontal tear in the posterior horn. Partial resection was performed again into a functional residual rim of 8 mm (Figure 5). After surgery the patient reported complete relief of symptoms. Physical therapy was begun immediately with the same protocol. The patient returned to sports activities without any restriction after 3 months.

We are still following-up the patient. At the time this manuscript was submitted he was 15 years old and 18 months had been elapsed since the second operation, and reportedly doing well.

There are several issues to consider in the case presented. Why did the lateral meniscus re-grow after surgery? Why did it return to its original incomplete discoid shape? Why was the patient symptomatic only on the right side?

Menisci in children have an increased vascularity and cellularity that are progressively lost with aging. They can be found throughout the inner parts of the menisci in patients aged 10 to 11 years [4]. Furthermore, during the growth spurt there may be some influence of growth and maturation of all tissues, including menisci. To the best of our knowledge there are no reports in literature about the vascularization of the inner part of discoid lateral meniscus in children, but we assume it is similar to a normal meniscus of the same age. During the 29 months that elapsed between first and second arthroscopy, our patient had undergone significant physical growth which may have had an impact on meniscal regeneration. One may argue that the surgeon did not resect enough of the meniscus during first arthroscopy. Surgeons treating meniscal lesions in children are concerned about removing too much tissue, because this could promote degenerative osteoarthritis. On the other hand, the aim of surgery in discoid meniscus is to restore its crescent shape. In our patient, the resection performed during the first operation was judged adequate (Figure 3) by the performing surgeon who is trained in pediatric knee arthroscopy, also demonstrated by the lack of symptoms referred by the patient during very active sport activities sustained in the period of 29 months between the first and the second arthroscopy.

The growth of the patient involves all structures of the knee and likely the lateral menisci. Normally the proportion and the shape of menisci are maintained from the fetal phase to adult age [4], but in our patient after surgery the right lateral meniscus re-created the previous condition of an incomplete discoid shape, as this would be its natural shape.

There is still debate about the etiology of discoid meniscus. Smillie [5] first proposed that this condition is the persistence of the normal stage during fetal development. However, most of the authors believe it is anomalous also during prenatal development, and arises through variant morphogenesis [4,6]. On the other hand, comparative data favor a phylogenetic origin because it represents, at least in some cases, the persistence of an ancestral character [7]. We believe that the re-growth of the discoid lateral meniscus in our patient favors the hypothesis of variant morphogenesis. In consideration of this condition, it is also often associated to other musculoskeletal abnormalities [1].

Also the left lateral meniscus was discoid-shaped, but the patient did not report any complaints on that side. In fact, stable discoid menisci are often an incidental finding and commonly asymptomatic [1].

## Conclusion

This case report demonstrates discoid meniscal re-growth in a child. The growth spurt may have an impact on meniscal regeneration. Re-growth of the discoid lateral meniscus in our patient favors the hypothesis of variant morphogenesis.

## Consent

Written informed consent was obtained from the parents of the young patient for publication of this Case report and any accompanying images. A copy of the written consent is available for review by the Editor of this journal.

## Competing interests

The authors declare that they have no competing interests.

## Authors’ contributions

SB: 1) had made substantial contributions to acquisition, analysis and interpretation of data; and 2) had been involved in drafting the manuscript. CT: 1) had made substantial contributions to conception and design; 2) had been involved in revising the manuscript critically for important intellectual content; and 3) had given final approval of the version to be published.

## Pre-publication history

The pre-publication history for this paper can be accessed here:

http://www.biomedcentral.com/1471-2474/14/285/prepub
